# Supplementary material for: A SIMPLI (Single-cell Identification from MultiPLexed Images) approach for spatially-resolved tissue phenotyping at single-cell resolution
Source: Nat Commun. 2022 Feb 9;13:781. doi: 10.1038/s41467-022-28470-x (PMC8828885; doi:10.1038/s41467-022-28470-x)
Supplement: Supplementary file 3 — Description of Additional Supplementary Files [file 41467_2022_28470_MOESM3_ESM.pdf]

Description of Additional Supplementary Files

File Name: Supplementary Data 1
Description: Samples used in the study

File Name: Supplementary Data 2
Description: Antibodies used in the study
