# Supplementary material for: A SIMPLI (Single-cell Identification from MultiPLexed Images) approach for spatially-resolved tissue phenotyping at single-cell resolution
Source: Nat Commun. 2022 Feb 9;13:781. doi: 10.1038/s41467-022-28470-x (PMC8828885; doi:10.1038/s41467-022-28470-x)
Supplement: Supplementary file 6 — Reporting Summary [file 41467_2022_28470_MOESM6_ESM.pdf]

Corresponding author(s): Francesca D. Ciccarelli

Last updated by author(s): Jan 3, 2022

Reporting Summary

Nature Research wishes to improve the reproducibility of the work that we publish. This form provides structure for consistency and transparency in reporting. For further information on Nature Research policies, see our Editorial Policies and the Editorial Policy Checklist.

Statistics

For all statistical analyses, confirm that the following items are present in the figure legend, table legend, main text, or Methods section.

n/a	Confirmed	
☐	☒	The exact sample size (*n*) for each experimental group/condition, given as a discrete number and unit of measurement
☒	☐	A statement on whether measurements were taken from distinct samples or whether the same sample was measured repeatedly
☐	☒	The statistical test(s) used AND whether they are one- or two-sided<br>*Only common tests should be described solely by name; describe more complex techniques in the Methods section.*
☒	☐	A description of all covariates tested
☐	☒	A description of any assumptions or corrections, such as tests of normality and adjustment for multiple comparisons
☐	☒	A full description of the statistical parameters including central tendency (e.g. means) or other basic estimates (e.g. regression coefficient) AND variation (e.g. standard deviation) or associated estimates of uncertainty (e.g. confidence intervals)
☐	☒	For null hypothesis testing, the test statistic (e.g. *F*, *t*, *r*) with confidence intervals, effect sizes, degrees of freedom and *P* value noted<br>*Give P values as exact values whenever suitable.*
☒	☐	For Bayesian analysis, information on the choice of priors and Markov chain Monte Carlo settings
☒	☐	For hierarchical and complex designs, identification of the appropriate level for tests and full reporting of outcomes
☐	☒	Estimates of effect sizes (e.g. Cohen's *d*, Pearson's *r*), indicating how they were calculated

Our web collection on statistics for biologists contains articles on many of the points above.

Software and code

Policy information about availability of computer code

Data collection	Data collection software used in this study:<br>Inform v2.5.0<br>imctools v1.0.7
Data analysis	Data analysis software used in this study:<br>Inform v2.5.0<br>CellProfiler v4.2.1<br>scikit-image v0.18.3<br>StarDist v0.7.3<br>EBImage v.4.35.2<br>Seurat v.2.3.0<br>fpc v2.2.8<br><br>The code for the software employed in the study is deposited at: https://github.com/ciccalab/SIMPLI (https://doi.org/10.5281/zenodo.5807230)<br>Custom scripts are described in the text, and are available upon request.

For manuscripts utilizing custom algorithms or software that are central to the research but not yet described in published literature, software must be made available to editors and reviewers. We strongly encourage code deposition in a community repository (e.g. GitHub). See the Nature Research guidelines for submitting code & software for further information.

Data

Policy information about availability of data

All manuscripts must include a data availability statement. This statement should provide the following information, where applicable:

- Accession codes, unique identifiers, or web links for publicly available datasets
- A list of figures that have associated raw data
- A description of any restrictions on data availability

The imaging mass cytometry data of human colon mucosa generated in this study have been deposited in the Zenodo database under accession code 5545882 [https://doi.org/10.5281/zenodo.5545882]{Bortolomeazzi, 2021 #80}. The imaging mass cytometry data of human appendix generated in this study have been deposited in the Zenodo database under accession code 5545760 [https://doi.org/10.5281/zenodo.5545760]{Bortolomeazzi, 2021 #81}. The multiplex immunofluorescence data of human colorectal cancer generated in this study have been deposited in the Zenodo database under accession code 5545864 [https://doi.org/10.5281/zenodo.5545864]{Bortolomeazzi, 2021 #82}.Source data are provided with this paper.

Field-specific reporting

Please select the one below that is the best fit for your research. If you are not sure, read the appropriate sections before making your selection.

☒ Life sciences ☐ Behavioural & social sciences ☐ Ecological, evolutionary & environmental sciences

For a reference copy of the document with all sections, see nature.com/documents/nr-reporting-summary-flat.pdf

Life sciences study design

All studies must disclose on these points even when the disclosure is negative.

Sample size

No sample size calculation was performed. Sample sizes were chosen as the minimum required for each of the three case studies we use to demonstrate the features of the image analysis pipeline we present in the manuscript. For the analysis in figure 1 no comparisons across samples were made and six samples were sufficient to verify the correlation between IgA+ plasma cells in the lamina propria and secreted IgA in the epithelial compartment. For the analyses in figures 3, 4 and supplementary figures S2 and S3 no comparisons across samples were made and thus one image from one sample was sufficient. For figure 5 a preexistent dataset was used to replicate the results of a previous study.

Christian M. Schürch, Salil S. Bhate, Graham L. Barlow, Darci J. Phillips, Luca Noti, Inti Zlobec, Pauline Chu, Sarah Black, Janos Demeter, David R. McIlwain, Shigemi Kinoshita, Nikolay Samusik, Yury Goltsev, Garry P. Nolan, Coordinated Cellular Neighborhoods Orchestrate Antitumoral Immunity at the Colorectal Cancer Invasive Front, Cell, Volume 182, Issue 5, 2020, Pages 1341-1359.e19, ISSN 0092-8674, https://doi.org/10.1016/j.cell.2020.07.005.

Data exclusions

No data was excluded from the analysis.

Replication

No replication was required as the study does not report any experimental findings.

Randomization

No randomization or controlling of covariates was required in this study because no comparisons across samples group were made apart from the reanalysis of previous data from Schürch et. al which were were selected at random, matched for gender, age, and cancer type, location, and cancer stage in their original publication.

Christian M. Schürch, Salil S. Bhate, Graham L. Barlow, Darci J. Phillips, Luca Noti, Inti Zlobec, Pauline Chu, Sarah Black, Janos Demeter, David R. McIlwain, Shigemi Kinoshita, Nikolay Samusik, Yury Goltsev, Garry P. Nolan, Coordinated Cellular Neighborhoods Orchestrate Antitumoral Immunity at the Colorectal Cancer Invasive Front, Cell, Volume 182, Issue 5, 2020, Pages 1341-1359.e19, ISSN 0092-8674, https://doi.org/10.1016/j.cell.2020.07.005.

Blinding

No blinding was required in this study as all samples were analyzed with the same automated pipeline with the same parameters. Additionally no comparisons across samples group were made apart from the reanalysis of previous data from Schürch et. al.

Christian M. Schürch, Salil S. Bhate, Graham L. Barlow, Darci J. Phillips, Luca Noti, Inti Zlobec, Pauline Chu, Sarah Black, Janos Demeter, David R. McIlwain, Shigemi Kinoshita, Nikolay Samusik, Yury Goltsev, Garry P. Nolan, Coordinated Cellular Neighborhoods Orchestrate Antitumoral Immunity at the Colorectal Cancer Invasive Front, Cell, Volume 182, Issue 5, 2020, Pages 1341-1359.e19, ISSN 0092-8674, https://doi.org/10.1016/j.cell.2020.07.005.

Reporting for specific materials, systems and methods

We require information from authors about some types of materials, experimental systems and methods used in many studies. Here, indicate whether each material, system or method listed is relevant to your study. If you are not sure if a list item applies to your research, read the appropriate section before selecting a response.

Materials & experimental systems

n/a	Involved in the study
	[x] Antibodies
[x]	Eukaryotic cell lines
[x]	Palaeontology and archaeology
[x]	Animals and other organisms
	[x] Human research participants
[x]	Clinical data
[x]	Dual use research of concern

Methods

n/a	Involved in the study
[x]	ChIP-seq
[x]	Flow cytometry
[x]	MRI-based neuroimaging

Antibodies

Antibodies used

anti-CD45, Fluidigm, Cat Num: 3152016D, CD45-2B11, 1:500
anti-CD20, Fluidigm, Cat Num: 3161029D, H1, 1:250
anti-IgA, NovusBio, Cat Num: NB500-469, AD3, 1:100
anti-IgM, NovusBio, Cat Num: NBP2-34254, IM373, 1:200
anti-CD27, Fluidigm, Cat Num: 3171024D, EPR8569,1:300
anti-CD45RA, Fluidigm, Cat Num: 3166028D, HI100, 1:2000
anti-CD45RO, Fluidigm, Cat Num: 3173016D, UCHL1, 1:500
anti-CD4, Fluidigm, Cat Num: 3156033D, EPR6855, 1:200
anti-CD8, Fluidigm, Cat Num: 3162035D, D8A8Y, 1:800
anti-PD1, Fluidigm, Cat Num: 3165039D, EPR4877(2), 1:50
anti-CD3, Fluidigm, Cat Num: 3170019D, Polyclonal C-Terminal, 1:800
anti-FOXP3, Fluidigm, Cat Num: 3155016D, 236A/E7, 1:200
anti-CD68, Fluidigm, Cat Num: 3159035D, KP1, 1:400
anti-CD16, Fluidigm, Cat Num: 3146020D, EPR16784, 1:200
anti-CD11c, Abcam, Cat Num: ab216655, EP1347Y, 1:400
anti-PD-L1, RnD System, Cat Num: MAB1561, 130021, 1:70
anti-CD34, Abcam, Cat Num: ab213058, HPCA1/1171, 1:150
anti-Pan-keratin, Fluidigm, Cat Num: 3148020D, C11, 1:3000
anti-E-Cadherin, Fluidigm, Cat Num: 3158029D, 24E10, 1:3000
anti-Collagen type IV, NovusBio Cat Num: NBP1-97716, 1043, 1:30
anti-Ki67, Fluidigm, Cat Num: 3168022D, B56, 1:400
anti-Vimentin, Fluidigm, Cat Num: 3143029D, RV202, 1:8000
anti-SMA, Fluidigm, Cat Num: 3141017D, 1A4, 1:4000
anti-CAMK4, NovusBio, Cat Num: NBP2-37428, 8C5B8, 1:250
anti-IFNA5, CloudClone, Cat Num: MAG975Hu22, C1, 1:100
anti-VEGFC, Abcam, Cat Num: ab191274, 197CT7.3.4, 1:600
anti-CD3, Dako, Cat Num: A0452, Polyclonal, 1:200
anti-Rabbit-HRP, Dako, Cat Num: P0448, Polyclonal, 1:200
anti-CD8, Cell Signaling Technologies, Cat Num: 85336, Polyclonal, 1:200
anti-Granzyme B, Abcam, Cat Num: ab208586, D8A8Y, 1:100
anti-PD1, Abcam, Cat Num: ab137132, EPR20129-217, EPR4877(2), 1:300
anti-ki67, BD Biosciences, Cat Num: 550609, B56, 1:200
anti-CD68, Biolegend, Cat Num: 916104, KP1, 1:1000
anti-PD-L1, RnD System, Cat Num: MAB1561, 130021, 1:450

Validation

All antibodies are commercially available and validated:
All Fluidigm antibodies used in this study are Maxpar® antibodies developed and optimized for use with the Hyperion™ Imaging System with formalin-fixed, paraffin-embedded (FFPE) human tissue sections. Each lot of metal-conjugated antibody is quality control-tested on tissue sections using the Hyperion Imaging System and their staining patterns are verified by an independent pathologist.
The anti-IgM antibody was validated with Immunohistochemistry: on formalin-fixed, paraffin-embedded human Tonsil stained with igM Monoclonal Antibody (IM373)
The CD11c antibody (EP1347Y, ab216655) was validated by Immunohistochemistry (Formalin/PFA-fixed paraffin-embedded sections) analysis of Human tonsil tissue sections labeling CD11c with Purified ab52632 at 1:500 dilution (0.21 µg/ml). Heat mediated antigen retrieval was performed using ab93684 (Tris/EDTA buffer, pH 9.0). Tissue was counterstained with Hematoxylin. ImmunoHistoProbe one step HRP Polymer (ready to use) secondary antibody was used at 1:0 dilution. PBS instead of the primary antibody was used as the negative control. This data was developed using the same antibody clone in a different buffer formulation containing PBS, BSA, glycerol, and sodium azide (ab52632).
The PD-L1/B7-H1 antibody was detected in formalin fixed paraffin-embedded sections of human colon cancer using Mouse Anti-Human PD-L1/B7-H1 Monoclonal Antibody (Catalog # MAB1561) at 15 µg/mL overnight at 4 °C. Tissue was stained using the Anti-Mouse HRP-DAB Cell & Tissue Staining Kit (brown; Catalog # CTS002) and counterstained with hematoxylin (blue). Specific staining was observed in the cytoplasm.
The CD34 antibody was validated by Immunohistochemical analysis of formalin-fixed, paraffin-embedded Human angiosarcoma tissue labeling CD34 with ab213058 at 4 µg/ml.

The Collagen IV antibody was validated by Immunohistochemistry on a FFPE sample of human colon.
The CaMK4 antibody was validated by Immunohistochemistry: CaMKIV Antibody (8C5B8) [NBP2-37428] - Immunohistochemical analysis of paraffin-embedded rectum cancer tissues using CAMK4 mouse mAb with DAB staining.
The IFNA5 antibody was validated by DAB staining on IHC-P samples of human stomach tissue, human glioma, human breast cancer, and Human Liver Tissue.
The VEGFC antibody was validated by Immunohistochemical analysis of formalin-fixed, paraffin-embedded Human small intestine tissue labeling VEGFC with ab191274 at 1/50 dilution.
The anti-CD8 antibody from Cell Signaling Technologies was validated by Immunohistochemical analysis of paraffin-embedded human Crohn's diseased colon, lymphoma and lung carcinoma using CD8a (D8A8Y) Rabbit mAb.
The anti-GzB antibody from Abcam was validated by Immunohistochemical analysis of paraffin-embedded human colon tissue labeling Granzyme B with ab208586 at 1/250 dilution, followed by Goat Anti-Rabbit IgG H&L (HRP) (ab97051) at 1/500 dilution. Cytoplasmic staining on some stromal cells of human colon is observed. Counter stained with Hematoxylin. Secondary antibody only control: Used PBS instead of primary antibody, secondary antibody is Goat Anti-Rabbit IgG H&L (HRP) (ab97051) at 1/500 dilution.
The anti-PD1 antibody Immunohistochemistry (Formalin/PFA-fixed paraffin-embedded sections) analysis of human tonsil tissue labeling PD1 with purified ab137132 at 1/500. Heat mediated antigen retrieval was performed using Tris/EDTA buffer pH 9. ab97051, an HRP-conjugated goat anti-rabbit IgG (H+L) was used as the secondary antibody (1/500). Negative control: Secondary antibody only using PBS instead of primary antibody. Counterstained with hematoxyl. Perform heat mediated antigen retrieval with Tris/EDTA buffer pH 9.0 before commencing with IHC staining protocol.
The anti-PD1 antibody from Abcam PD-L1/B7-H1 in Human Colon Cancer. PD-L1/B7-H1 was detected in formalin fixed paraffin-embedded sections of human colon cancer using Mouse Anti-Human PD-L1/B7-H1 Monoclonal Antibody (Catalog # MAB1561) at 15 µg/mL overnight at 4 °C. Tissue was stained using the Anti-Mouse HRP-DAB Cell & Tissue Staining Kit (brown; Catalog # CTS002) and counterstained with hematoxylin (blue). Specific staining was observed in the cytoplasm.
The anti-Ki67 antibody from BDbiosciences was validated by immunohistochemistry on normal human tonsil.
The anti-CD68 antibody (clone KP1) from BioLegend was tested by IHC on FFPE tissue from human tonsil and Parkinson´s and Alzheimer´s disease human brain tissue. Following antigen retrieval using Sodium Citrate H.I.E.R., the tissue was incubated with the primary antibody at 5 µg/ml overnight at 4°C. BioLegend's Ultra Streptavidin (USA) HRP Detection Kit (Multi-Species, DAB, Cat. No. 929901) was used for detection followed by hematoxylin counterstaining, according to the protocol provided. Images were captured with a 40X objective. Scale Bar: 20 µm.

Human research participants

Policy information about studies involving human research participants

Population characteristics	Eight unrelated patients who underwent surgery for the removal of colorectal cancers. The patients are both male or female, and both microsateelite stable or unstable (assessment by immunoistochemistry). The mean age at diagnosis is 50.0 ± 18.5. and pathological tumour stage range from T2 to T4a. These characteristics are reported in full in Supplementary Data 1 and the Source Data.
Recruitment	No patient was recruited specifically for this study.
Ethics oversight	All patients provided written informed consent in accordance with approved institutional guidelines:<br>University College London Hospital, REC Reference: 20/YH/0088<br>Istituto Clinico Humanitas, REC Reference: ICH/25/09

Note that full information on the approval of the study protocol must also be provided in the manuscript.
